# Fabrication of PVA Coatings Applied to Electrospun PLGA Scaffolds to Prevent Postoperative Adhesions

**DOI:** 10.3390/jfb16020057

**Published:** 2025-02-10

**Authors:** Arsalan D. Badaraev, Evgenii V. Plotnikov, Vladislav R. Bukal, Gleb E. Dubinenko, Johannes Frueh, Sven Rutkowski, Sergei I. Tverdokhlebov

**Affiliations:** 1 Weinberg Research Center, School of Nuclear Science and Engineering, National Research Tomsk Polytechnic University, 30, Lenin Avenue, 634050 Tomsk, Russia; adb6@tpu.ru (A.D.B.); vrb2@tpu.ru (V.R.B.); dubinenko@tpu.ru (G.E.D.); johannes.frueh@alumni.ethz.ch (J.F.); 2 Research School of Chemistry & Applied Biomedical Sciences, Tomsk Polytechnic University, 634000 Tomsk, Russia; plotnikovev@tpu.ru; 3 Mental Health Research Institute, Tomsk National Research Medical Center of the Russian Academy of Sciences, Aleutskaya Street, 634014 Tomsk, Russia

**Keywords:** electrospinning, poly(lactide-co-glycolide), polyvinyl alcohol, dip-coating, anti-adhesive properties, biomedical application

## Abstract

There is currently a demand for anti-adhesive materials that are capable of preventing the formation of intra-abdominal adhesions. In this study, electrospun poly(lactide-co-glycolide) scaffolds were dip-coated in aqueous solutions of polyvinyl alcohol with concentrations of 3 wt.%, 6 wt.% and 9 wt.% to obtain a nontoxic and anti-adhesive biomedical material. The viscosities of the applied 3 wt.%, 6 wt.% and 9 wt.% polyvinyl alcohol solutions were 7.7 mPa∙s, 38.2 mPa∙s and 180.8 mPa∙s, respectively, and increased exponentially. It is shown that increasing the viscosity of the polyvinyl alcohol solution from 6 wt.% to 9 wt.% increases the thickness of the polyvinyl alcohol layer from (3.32 ± 0.97) µm to (8.09 ± 1.43) µm. No pronounced polyvinyl alcohol layer can be observed on samples dip-coated in 3 wt.% PVA solution. Increasing the viscosity of the polyvinyl alcohol solution from 3 wt.% to 9 wt.% increases the mechanical properties of the poly(lactide-co-glycolide) samples by a factor of 1.16–1.45. Cytotoxicity analysis of all samples reveals that none is toxic to 3T3-L1 fibroblast cells. A cell adhesion assay indicates that the anti-adhesion properties increase with increasing viscosity of the polyvinyl alcohol solution and the thickness of the polyvinyl alcohol layer on the poly(lactide-co-glycolide) scaffolds. Fluorescence images of the cells show that as the thickness of the polyvinyl alcohol coating increases, the number of cells decreases, and they do not cover the surface of the samples and form spherical three-dimensional agglomerates. The highest mechanical and anti-adhesion properties are obtained with the poly(lactide-co-glycolide) scaffold sample dip-coated in the 9 wt.% polyvinyl alcohol solution. This is because this sample has the thickest polyvinyl alcohol coating.

## 1. Introduction

Mechanical trauma, inflammatory processes in the abdominal cavity and postoperative complications can lead to the development of fibrous, connective tissue, resulting in intra-abdominal adhesions [1]. Postoperative complications in the form of abdominal adhesions occur in more than 50% of cases following surgical procedures [2]. According to the literature, adhesions occur in 54% of cases after abdominal surgeries, in 66% after gastrointestinal surgeries and in 51% after obstetric surgeries [3]. The formation of adhesions in the abdominal region significantly reduces the quality of life of patients, causing them chronic pain, intestinal obstruction, female infertility, etc. [4].

Various electrospun polymeric materials are used to prevent intra-abdominal adhesions [5]. For example, electrospun polyester membranes with antibacterial and anti-adhesive properties have been fabricated [6]. Multilayered electrospun aliphatic polyester scaffolds with ibuprofen have been prepared, which exhibit high anti-adhesive properties towards L-929 fibroblasts [7]. Moreover, cross-linked aliphatic polyester scaffolds with acellular cartilage matrix have been fabricated, which exhibit anti-adhesive properties towards human umbilical vein endothelial cells [8]. In particular, electrospun poly(lactide-co-glycolide) (PLGA) scaffolds are often used to prevent tissue adhesion [9,10]. For example, electrospun nonwoven scaffolds have been prepared from PLGA, which possess anti-adhesive properties in vivo [11]. Even the combination of anti-adhesive electrospun PLGA nanofibrous membranes containing growth factors and drugs shows promising results in in vivo experiments on Wistar rats [12]. However, nonwoven PLGA scaffolds themselves do not have high anti-adhesive properties in vitro, as the nonwoven structure promotes cell adhesion, proliferation and migration [13,14]. In addition, electrospun PLGA scaffolds have poor mechanical properties (ultimate tensile strength might not exceed approximately 3 MPa) [15,16], which increases the risks of integrity loss after transplantation.

To address these problems, smooth coatings of biodegradable and biocompatible polymers can be applied to inhibit cell overgrowth [17] and enhance the mechanical properties of the scaffolds [18,19]. Among the many different materials with anti-adhesive properties, polyvinyl alcohol (PVA) is particularly noteworthy. This polymer is widely used for biomedical purposes due its biodegradable, biocompatible, nontoxic and hydrophilic properties [20]. PVA is currently used to make thin films [21], electrospun scaffolds or membranes [22,23] and hydrogels [24,25] with anti-adhesive properties. PVA has barrier properties against small molecules (oxygen, carbon dioxide, etc.) [26,27]. In addition, hydrophilic polymers can inhibit protein adsorption by forming a hydration layer [28,29], which is crucial for cell attachment [30]. PVA films have high mechanical properties, with reported maximum tensile strength and maximum elongation at break of 28 MPa and 205%, respectively [31]. Compared to other polymers used for the fabrication of medical materials with anti-adhesive properties (PVDF, polyurethane) [32], PVA is biodegradable, and its decomposition products are less toxic than PVA itself [33].

Various techniques and methods are used for the deposition of polymer-based coatings for biomedical applications: spin coating [34], spray coating [35], solvent casting [36], dip-coating [37,38], etc. Compared to spin coating and spray coating, the dip-coating process is one of the simplest methods that does not require significant resources and specialized equipment [39]. Compared to solvent casting, substrates of almost any shape can be coated using the dip-coating method [40].

Thus, the aim of this work is to prepare PVA coatings on electrospun PLGA scaffolds to improve their mechanical and anti-adhesion properties for further use as barrier membranes to prevent postoperative adhesions in the abdominal cavity. To fabricate PVA coatings, a dip-coating method will be used, because of its simplicity and ability to coat substates of practically any form. This work focuses on investigation of the morphological, physico-chemical, cytotoxic and cell anti-adhesive properties of the uncoated and PVA-coated PLGA scaffolds.

## 2. Materials and Methods

### 2.1. Preparation of Electrospun PLGA Scaffolds

First, granules of poly(L-lactide-co-glycolide) (PLGA 85/15, Mn ≈ 202,000 g/mol, Corbion Purac, Amsterdam, The Netherlands) were dissolved in hexafluoroisopropanol (HFIP, (CF_3_)_2_CHOH, P&M Invest, Moscow, Russia) to obtain a 4 wt.% polymer solution. The prepared solutions were then fed into an electrospinning machine (NANON-01A, MECC Co., Fukuoka, Japan) equipped with a rotating cylindrical collector with a length of 200 mm and a diameter of 100 mm. For the electrospinning process, the following technological parameters were applied: voltage—+22 kV, polymer solution feed rate—4 mL/h, needle-tip-to-collector distance—150 mm, rotational speed of the collector—50 rpm, chamber temperature—(26.2 ± 4.5) °C, humidity in the electrospinning chamber—(19 ± 6)%. In order to evaporate the residual solvent in the PLGA scaffolds after preparation, the scaffolds were removed from the collector and placed in a vacuum chamber at a pressure of 100 Pa for 24 h. A more detailed description of the technological parameters used for electrospinning and why these parameters were chosen for this study can be found in reference [41].

### 2.2. Preparation of PVA Solutions and Viscosity Measurements

Polyvinyl alcohol powder (PVA, Mw = 85,000–124,000 g/mol, Sigma Aldrich, St. Louis, MO, USA) were dissolved in distilled water (OOO «Gelena Chimavto», Moscow, Russia) with an electrical conductivity at 25 °C not higher than 5.1 × 10^−4^ S/m to obtain solutions with a PVA concentration of 3, 6 and 9 wt.%. To make solutions homogeneous, the PVA solutions were placed in a simple water bath heated to 90 °C for 2 h. The dynamic viscosity of the homogenous PVA solutions was measured at a temperature of (24.5 ± 0.4) °C using a viscometer (SV-10, AND, Tokyo, Japan).

### 2.3. PVA Coating Application by Dip-Coating

The prepared 3, 6 and 9 wt.% PVA solutions were poured into Petri dishes with an outer diameter of 10 cm up to half of the total volume of the dish. Then, 5 × 5 cm^2^ PLGA samples were immersed in the Petri dishes with the PVA solutions in such a way that the samples were completely immersed in PVA solutions. One side of the samples was immersed in a Petri dish and soaked in PVA solutions for one minute, and then the samples were turned over and soaked for one another minute. The samples soaked with the PVA solutions were attached to a taut rope and dried overnight under room conditions. To remove the remaining water, the samples were then placed in a vacuum chamber (Actan Vacuum, VTSH-K24-250-P, Moscow, Russia) at room temperature and a pressure of 100 Pa for 24 h.

### 2.4. Mass and Thickness Analysis

The mass and thickness of the samples were analyzed before and after immersion in PVA solutions. For this purpose, a laboratory scale (Acculab ALC-210d4, Sartorius AG, Göttingen, Germany) and a thickness gauge (TN 10-60 M, OOO NPO Kirov Instrument, Kirov, Russia) were used. The thickness and mass of each sample type were measured with five samples.

### 2.5. Optical Microscopy of Sample Surfaces and Their Cross-Sections

An optical bright-field microscope (DM-1802 Motic, Xiamen, Fujian, China) equipped with 10×, 20× and 40× objectives was used to obtain optical micrographs of the unmodified and dip-coated PLGA scaffold surfaces to evaluate their morphology. To measure the average thickness of fibers of each sample’s surfaces, optical bright-field microscopy images with 40× objectives were taken. For the measurements, the micrographs were processed using ImageJ software (version 1.51w, National Institutes of Health, Washington, DC, USA), and approximately 200 fiber diameter values were determined for each sample.

To analyze the thickness of the PLGA scaffold samples and the thickness of the PVA layers on the scaffolds, cross-sectional bright-field micrographs were taken with 10× and 20× objectives. The thickness values were calculated from the micrographs using ImageJ software (version 1.51w, National Institutes of Health, Washington, DC, USA), and for each sample approximately 50 values were determined.

### 2.6. Scanning Electron Microscopy

The morphology of the unmodified and dip-coated scaffold surfaces was investigated using a scanning electron microscope (SEM; ESEM Quanta 200 3D, FEI Company, Hillsboro, OR, USA) at magnifications of 1000× and 5000× and an accelerating voltage of 20 kV in low vacuum mode. Mean fiber diameter values were determined from SEM micrographs at 5000× magnification using ImageJ software (version 1.51w, National Institutes of Health, Washington, DC, USA) by obtaining at least 200 fiber diameter values for each sample.

### 2.7. Optical Profilometry

Interferometric surface relief and mean roughness depth (R_z_) of unmodified and PVA-coated electrospun PLGA scaffolds were evaluated using an optical profilometer (MNP-1, Technological Design Institute of Scientific Instrument Engineering SB RAS, Novosibirsk, Russia). The following parameters were used for the measurements: scanning range—up to 40 µm, scanning step—0.5 µm, minimum threshold for detection—2.7.

### 2.8. Porosity of the Scaffold Samples

For porosity analysis, the gravimetric method was used to calculate the porosity values (*P*) of the samples. These porosity values were calculated using the following equation [42]:

(1) $$P=(1-\frac{\rho_{scaffold}}{\rho_{solid}})\cdot 100\%,$$

where ρ_scaffold_ is the density of uncoated and PVA coated scaffolds in g/cm^3^, and ρ_solid_ is the density of uncoated and surface-modified samples consisting of PLGA and PVA. This density was calculated using the following equation:

(2) $${\rho_{solid}=k}_{PVA}\cdot \rho_{PVA}+k_{PLGA}\cdot \rho_{PLGA},$$

where ρ_PVA_ and ρ_PLGA_ are the densities of bulk PVA (1.25 g/cm^3^) [43] and bulk PLGA (1.25 g/cm^3^), respectively [44]; and k_PVA_ and k_PLGA_ are the mass fractions of PVA and PLGA, respectively, in coated scaffolds.

### 2.9. Elemental Composition by Energy-Dispersive X-Ray Spectroscopy Analysis

The elemental composition analysis of the unmodified and dip-coated scaffolds was carried out using an energy-dispersive X-ray spectrometer (EDX, Genesis 4000, Oxford Instruments, Abingdon, UK), integrated into an SEM (ESEM Quanta 200 3D, FEI Company, Hillsboro, OR, USA) at magnification of 1000×. For the analysis, a ZAF-correction (Z—atomic number, A—absorption effect and F—fluorescence excitation effect) was applied.

### 2.10. Fourier-Transform Infrared Spectroscopy

The chemical composition of the unmodified and dip-coated PLGA scaffolds was investigated by Fourier-transform infrared spectroscopy (FTIR) utilizing a spectrometer (Agilent Cary 630, Agilent, Santa Clara, CA, USA). FTIR spectra were recorded in the wavenumber range of (4000–650) cm^−1^ with a resolution of 0.93 cm^−1^.

### 2.11. X-Ray Diffraction (XRD)

The crystallinity of the samples prepared was analyzed using an X-ray diffractometer (XRD 6000, Shimadzu, Kyoto, Japan). A direct beam with 2θ in the range of (10–80)° with an X-ray tube voltage of 40 kV, a current of 30 mA and a scanning speed of 2 °/min was used for all samples. The crystallite size (*CS*) was calculated according to the following equation [45]:

(3) $$CS=\frac{0.9\lambda }{\beta \;cos\theta },$$

where *λ* is the wavelength of the Cu Ka radiation in the X-ray tube (equals 1.54 Å), *β* is the half-width of the diffraction peak, measured in °, and *θ* is the diffraction angle in °.

The crystallinity degree (*CD*) was calculated as the ratio of the areas of all crystalline peaks (*C*) to the area of all crystalline (*C*) and amorphous (*A*) peaks [46]:

(4) $$CD=\frac{C}{C+A}$$

### 2.12. Thermogravimetric Analysis

A thermal analyzer (SDT-Q600, TA Instruments, New Castle, DE, USA) was used for thermogravimetric analysis of the samples. A square with an area of 1.5 × 1.5 cm^2^ was cut from each sample. The prepared squares were placed in a heat-resistant vessel, which was placed in a thermal analyzer, and then the temperature was increased from room temperature to 800 °C at a heating rate of 20 °C/min under air. Finally, thermogravimetric (TG) and differential thermogravimetric (DTG) curves were obtained.

### 2.13. Wettability of the Samples

To determine the wettability of the samples, the water contact angles (WCAs) were measured using a drop shape analyzer (DSA 20, Krüss, Hamburg, Germany). The WCAs were measured 2, 30 and 60 s after the interaction of the droplets with the sample surfaces. Three water droplets of 2 μL each were deposited on the surface of each distinct sample with an area of 3 × 1 cm^2^.

### 2.14. Mechanical Properties

The analysis of tensile strength, maximum elongation at break and Young’s modulus was performed on a tensile testing machine (Instron 3343, Illinois Tool Works, Glenview, IL, USA) with a static load cell of 50 N (Instron 2519-102, Illinois Tool Works, Glenview, IL, USA). For each scaffold sample, the size was 3 × 1 cm^2^, and its length was cut according to the direction of rotation of the cylindrical collector of the electrospinning device. The space between the traverse jaws was 1 × 1 cm^2^, and the traverse speed was 20 mm/min. All measurements were conducted in triplicate.

### 2.15. Cytotoxicity and Anti-Adhesive Properties

For analysis of the anti-adhesive properties and cytotoxicity of the samples under investigation, the mouse fibroblast cell line 3T3-L1 was used. The choice of the 3T3-L1 cell line for the evaluation of the anti-adhesive properties of the scaffold is well-justified for several reasons. Cells of the 3T3-L1 line exhibit fibroblast-like morphology and behavior in their undifferentiated state, making them an excellent model for connective tissue studies and scratch and biocompatibility tests [47]. These 3T3L1 fibroblasts are commonly used for such in vitro experiments [48], including adipocyte-related research, as their fundamental fibroblastic characteristics make them an appropriate and scientifically valid choice for evaluating scaffold adhesion properties in connective tissue engineering applications [49]. The 3T3-L1 cells maintain their fibroblastic phenotype unless they are specifically induced to differentiate into adipocytes through chemical stimulation [50]. These cells have been used in conjunction with in vivo studies to analyze the anti-adhesive properties of polymer films [51]. Their well-characterized behavior allows them to be used to assess the biocompatibility of different materials and the interactions between cells and scaffolds. The cells were obtained from the Institute of Cytology of the Russian Academy of Sciences (Institute of Cytology, Russian Academy of Sciences, St. Petersburg, Russia). This cell line has been extensively validated for the study of cell–material interactions in various scaffold compositions, including collagen, fibronectin and synthetic polymers. Cells were maintained in Dulbecco’s Modified Eagle Medium (DMEM, Gibco, Billings, MT, USA) supplemented with 10% fetal bovine serum, 2 mM L-glutamine, penicillin (50 IU/mL) and streptomycin (50 µg/mL). The cells were cultivated in a CO_2_ incubator (CB-170, Binder, Tuttlingen, Germany) in a humidified atmosphere containing 5% CO_2_ at 37 °C. Discs with a diameter of 10 mm each were used as samples, cut from the unmodified and dip-coated scaffold samples. Cell preparation, sample sterilization, cytotoxicity and the methodology of cell adhesion assay were performed according to reference [41], with the only difference that in this study the analysis was performed after 24 and 120 h of incubation. In brief, the sample discs were subjected to an extraction procedure to evaluate potential cytotoxicity. Individual discs were placed in 24-well plates (SPL Life Sciences Co., Ltd., Pocheon, Republic of Korea) and immersed in 1 mL of the DMEM medium per well. The extraction was conducted over a 5-day period at standard cell culture conditions (37 °C, 5% CO_2_, 95% humidity). Control wells containing 1 mL DMEM without sample discs were maintained under the conditions. To assess cytotoxicity, fibroblasts were seeded at a density of 3000 cells per well in a sterile 96-well plate (SPL Life Sciences Co., Ltd., Pocheon, Republic of Korea). Following the initial 24 h attachment period, the culture medium was replaced with 5-day DMEM medium extracts obtained from the disc samples. Cell viability was evaluated on days 1 and 5 using the standard MTT assay. The culture medium was replaced with 3-(4,5-dimethylthiazol-2-yl)-2,5-diphenyl-2H-tetrazolium bromide solution (MTT, PanEco Ltd., Moscow, Russia) at 0.45 mg/mL concentration. After four hours of incubation at 37 °C, the MTT solution was removed, and the formazan crystals were dissolved in 100 µL dimethyl sulfoxide (DMSO, PanEco Ltd., Moscow, Russia). The absorbance was measured at 570 nm using a microplate photometer (Multiskan FC, Thermo Fisher Scientific Inc., Waltham, MA, USA). Biocompatibility evaluation was conducted with scaffold disc samples placed in 24-well plates (SPL Life Sciences Co., Ltd., Pocheon, Republic of Korea). Fibroblasts were seeded onto the disc sample surfaces at a density of 5 × 10⁴ cells/mL. Cell growth and density were monitored at days 1 and 5 after seeding, using fluorescent staining. A mixture of the vital fluorescent dyes Calcein AM (0.5 µg/mL, Thermo Fisher Scientific, Waltham, MA, USA) and Hoechst 33342 (1 µg/mL, Lumiprobe Corporation, Hunt Valley, MD, USA) in a volume of 200 µL was added to each sample disc with cells growing on its surface. The visualization of the cells was performed using an inverted fluorescence microscope (Zeiss AxioVert A1, Carl Zeiss AG, Oberkochen, Germany). Quantification of the cells was carried out using ImageJ software (version 1.53v, National Institutes of Health, Bethesda, MD, USA). The results are expressed as the mean number of cells per 1.0 mm² of sample surface.

### 2.16. Statistics

Statistical data processing was performed using the OriginPro^®^ 2021 program (Origin-Lab, Northampton, MA, USA). Differences in fiber diameters, porosity, roughness, PVA layer thickness, mechanical properties, wettability, cytotoxicity and anti-adhesive properties of unmodified and dip-coated PLGA scaffolds were evaluated using the one-way ANOVA and Mann–Whitney U tests. The differences were statistically significant at *p* < 0.05.

**Figure 1:**
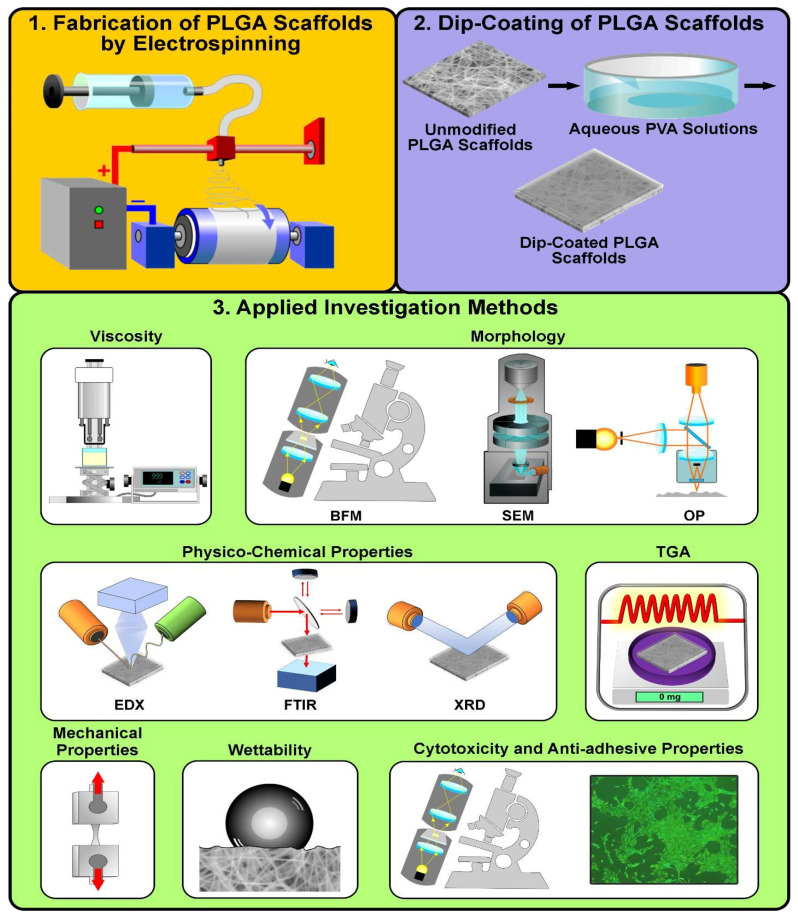
Schematic illustration of the preparation of poly(lactide-co-glycolide) (PLGA) scaffolds, their dip-coating in polyvinyl alcohol (PVA) and the investigation methods employed in this study. In the first stage, PLGA scaffolds were fabricated by electrospinning. At the second stage, the prepared PLGA scaffolds were dip-coated in aqueous solutions with PVA concentrations of 3 wt.%, 6 wt.% and 9 wt.%. Finally, the physico-chemical, morphological, cytotoxic and anti-adhesion properties of the samples prepared in this study were determined using the methods illustrated. The abbreviations used refer to the following: BFM—bright-field microscopy, SEM—scanning electron microscopy, OP—optical profilometry, EDX—energy-dispersive X-ray analysis, FTIR—Fourier-transform infrared spectroscopy, XRD—X-ray diffraction and TGA—thermal gravimetric analysis.

A schematic overview of the samples’ preparation, their modification with PVA coatings as well as the examination methods used is shown in Figure 1.

## 3. Results and Discussion

### 3.1. Viscosity of the PVA Solutions and Changes in Mass and Thickness of PLGA Scaffolds After Dip-Coating

The viscosity of PVA solutions with 3 wt.%, 6 wt.% and 9 wt.% in distilled water corresponds to (7.7 ± 0.5) mPa∙s, (38.2 ± 0.6) mPa∙s and (180.8 ± 2.0) mPa∙s (see Supplementary Materials (SI) Figure S1a). After dip-coating in 3 wt.%, 6 wt.% and 9 wt.% PVA solutions, the weight of the samples increased by (5.6 ± 0.8) mg—(8.8 ± 1.6) wt.%, (19.6 ± 6.5) mg—(34.8 ± 7.8) wt.% and (39.8 ± 2.1) mg—(79.2 ± 3.9) wt.%, respectively (see Supplementary Materials (SI) Figure S1b,c). The initial weights of the dip-coated PLGA scaffolds are given in see Supplementary Materials Table S1. With increasing viscosity of the PVA solutions, the mass of the samples increases logarithmically (see Supplementary Materials Figure S2a). As the concentration of PVA in the solution for dip-coating increases, the dynamic viscosity increases exponentially (see Supplementary Materials Figure S2b).

### 3.2. Surface Morphology of the PLGA Scaffolds

**Figure 2:**
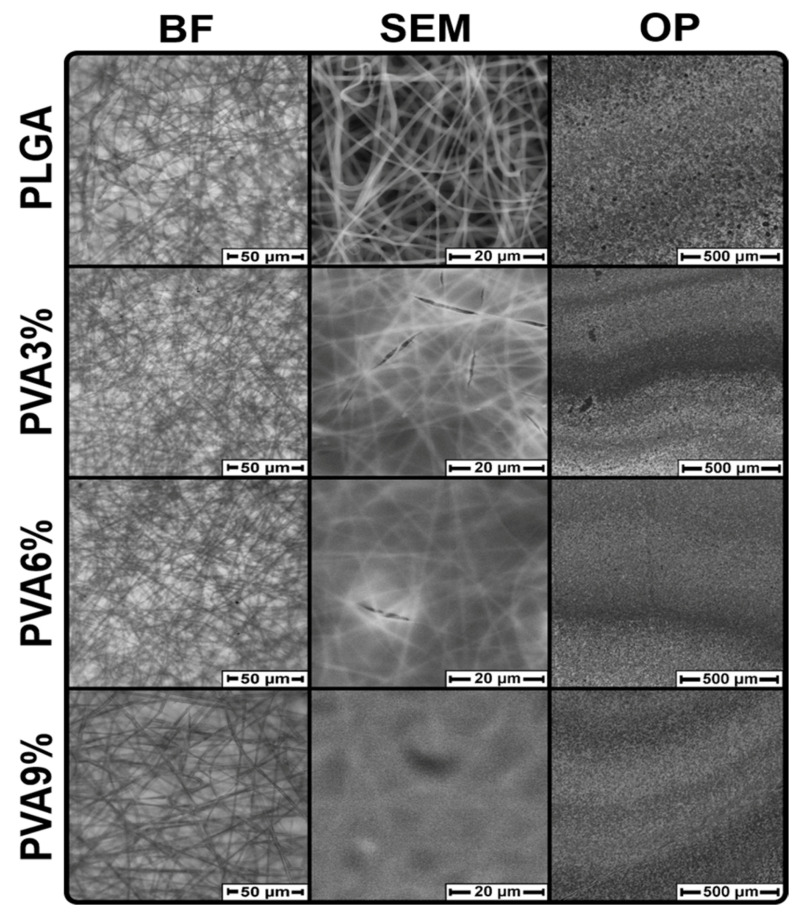
Morphology of PLGA scaffolds before (PLGA) and after dip-coating in three PVA solutions (PVA3%, PVA6%, PVA9%). On the left are bright-field (BF) micrographs, in the middle are scanning electron microscopy (SEM) micrographs at 5000× magnification and on the right are interferometric surface topography micrographs obtained by optical profilometry (OP). n = 5 for each microscopic method, whereby the micrographs displayed here are representative of the respective sample group.

The surface morphology of the PLGA scaffolds prepared before and after dip-coating in PVA solutions is displayed in Figure 2.

It can be observed from the micrographs that the PLGA scaffolds possess a nonwoven, fibrous morphology (Figure 2 and see Supplementary Materials Figure S3). The SEM micrographs reveal that a film has formed on the PLGA scaffolds after dip-coating in PVA solutions. For the PVA3% and PVA6% samples, this film has cracks, but the film in PVA9% samples has formed without cracks (Figure 2 and Figure S3). In the case of the PVA9% samples, the fibers are barely visible due to the PVA film formed. This indirectly indicates that the film thickness increases when the samples are dip-coated in more viscous solutions. The coating is transparent, such that individual fibers can be seen in the bright-field micrographs for all sample groups (Figure 2 and Figure S3). From the surface photographs obtained by optical profilometry (OP), it can be observed that the PLGA scaffold, which is not dip-coated in PVA solution, has a coarse grain structure, which is most likely related to the nonwoven morphology of the scaffolds. After dip-coating of the scaffolds in PVA solutions, their surface becomes smoother and less grainy (Figure 2, OP).

**Figure 3:**
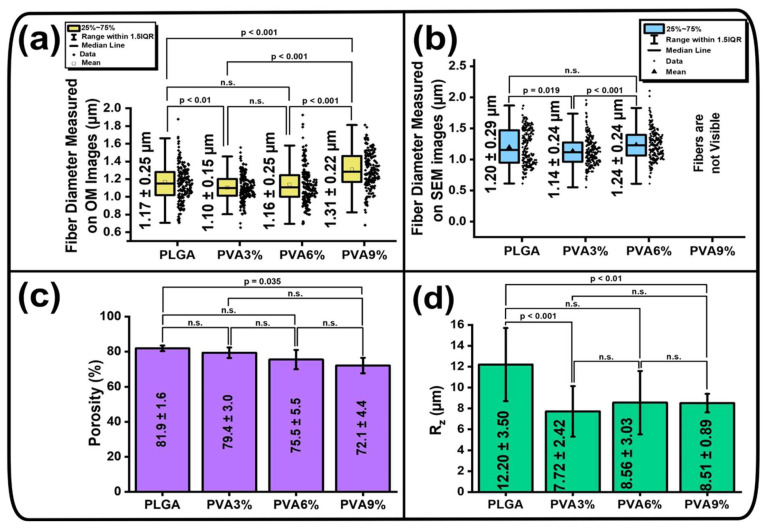
Values of the mean fiber diameter, porosity and average roughness depth (R_z_) of PLGA scaffolds before (PLGA) and after dip-coating (PVA3%, PVA6%, PVA9%): (**a**) mean fiber diameter values based on bright-field (BF) micrographs (mean ± standard deviation (M ± SD), n = 200); (**b**) mean fiber diameter values based on scanning electron microscopy (SEM) micrographs (M ± SD, n = 200); (**c**) porosity of the scaffolds, calculated using the gravimetric method (M ± SD, n = 3); (**d**) average roughness depth (R_z_), calculated from interferometric surface topography measurements (M ± SD, n = 6–10). Abbreviation “n.s.”—not statistically significant.

The mean fiber diameter values calculated from bright-field microscopy and scanning electron microscopy micrographs, the porosity values and the average roughness depth (R_z_) calculated from interferometric surface topography are presented in Figure 3.

Based on bright-field (BF) micrographs, the mean fiber diameter of all samples is in the range of ((1.17–1.31) ± 0.25) µm (Figure 3a). From the calculations of the scanning electron microscopy (SEM) micrographs, it can be seen that the mean fiber diameter of the PLGA samples is (1.20 ± 0.29) µm, for PVA3% it is (1.14 ± 0.24) µm, for the PVA6% samples the mean fiber diameter is (1.24 ± 0.24) µm, and for the PVA9% samples the individual fibers are barely visible (Figure 3b). After dip-coating of the PLGA scaffolds in an aqueous PVA solution, the diameter of PLGA fibers changes statistically, but only within a small range. (Figure 3a,b). This indicates that the initial morphology and structure of the individual PLGA fibers are preserved after the dip-coating process and that the PLGA fibers do not absorb the PVA. The porosity of PLGA scaffolds is (81.9 ± 1.6)%, while the porosity of PVA3% samples is (79.4 ± 3.0)%, of PVA6% samples is (75.5 ± 5.5)% and of PVA9% samples is (72.1 ± 4.4)% (Figure 3c). The values of porosity were mainly not statistically significant (n.s.), but the tendency of porosity decrease with increase of solution viscosity is still observed, as the statistical difference between unmodified and PVA9% samples is defined as *p* = 0.0035 (Figure 3c). The average roughness depth (R_z_) of the PLGA scaffolds is (12.2 ± 3.5) µm, while the values for the scaffolds which were dip-coated in PVA solutions are in the range of ((7.72–8.56) ± 3.03) µm (Figure 3d). It can be seen that there is no statistically significant difference between the PVA3%, PVA6% and PVA9% samples, but unmodified PLGA samples have a statistical difference with the dip-coated scaffolds, at *p* < 0.01 (Figure 3d). This indicates that the PLGA scaffold samples become smoother after dip-coating in PVA solutions.

### 3.3. Morphology of the PLGA Scaffold Cross-Sections

**Figure 4:**
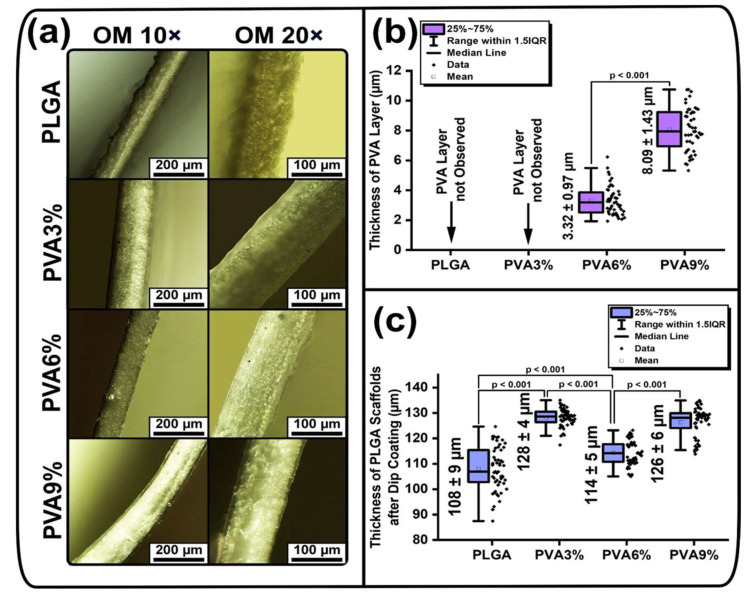
Cross-sectional bright-field micrographs, thicknesses of the PVA coating and the PLGA scaffolds of the samples before (PLGA) and after dip-coating (PVA3%, PVA6%, PVA9%): (**a**) cross-sectional micrographs taken with 10× and 20× objectives; (**b**) thickness of PVA layers on PLGA scaffolds (mean ± standard deviation (M ± SD), n = 50); (**c**) overall thickness of the samples (M ± SD, n = 50).

The cross-sectional bright-field micrographs obtained with 10× and 20× objectives and the thickness values of the PVA coating on PLGA scaffolds as well as the thicknesses of the uncoated and coated scaffolds are shown in Figure 4.

Cross-sectional micrographs of PLGA scaffolds show individual fibers protruding from them (Figure 4a). The cross-sectional micrographs of the samples dip-coated in PVA solutions demonstrate that the surface has a uniform smooth structure (Figure 4a). In the bright-field micrographs of PLGA and PVA3% scaffolds, the PVA layer is not clearly recognizable (Figure 4a). However, in the PVA6% samples, a thin layer of semi-transparent PVA coating can be seen on both sides of the PLGA scaffold, while in the PVA9% sample a thick transparent PVA layer is clearly visible (Figure 4a). The thickness of the PVA layer was estimated on the basis of the bright-field micrographs obtained. It can be seen that there is no distinct PVA layer on the PVA3% sample, which is why no thickness values are presented (Figure 4b). This could be due to the fact that PVA can penetrate into the nonwoven structure of PLGA at low viscosity and solution concentration and forms a uniform thin layer on the surface of the PLGA scaffolds with increasing viscosity (Figure 4a). The mean thickness of the PVA layer for PVA6% samples equals (3.32 ± 0.97) µm, and for PVA9% samples it is (8.09 ± 1.43) µm (Figure 4b). It is clearly visible that the PVA layer on the PLGA scaffolds becomes thicker as the concentration the PVA solutions increases, which is confirmed by the fact that the differences are statistically significant, at *p* < 0.001 (Figure 4b). The thickness of the investigated PLGA scaffolds before and after dip-coating is in the range of ((108–128) ± 9) µm (Figure 4c). All thickness values of the samples are statistically different, at *p* < 0.001 (Figure 4c). Due to the formation of the PVA layer, the thickness of the PLGA scaffolds is barely changed (Figure 4c). This might be due to the fact that the thickness of the PLGA scaffolds decreases after dip-coating, as the weight of the PVA layer can compress the fibers and thus decrease the scaffold thickness.

### 3.4. Elemental and Chemical Composition, Thermal Properties and Crystalline Structure of the PLGA Scaffolds

In the analysis of the elemental composition by the semi-quantitative EDX, it can be observed that as the mass of PVA on the scaffolds increases (SI Figure S1b,c and see Supplementary Materials Table S1), the carbon-to-oxygen ratio (C/O) increases by 1.31 to 1.70 times in wt.% and by 1.74 to 2.26 times in at.% (see Supplementary Materials Table S2). This indicates an increase in the PVA fraction in the PLGA scaffold samples, as commonly five oxygen atoms (if the ratio between lactic acid and glycolic acid is 1/1) are bound in a monomer unit of PLGA, while one oxygen atom is bound in the monomer unit of PVA.

The FTIR spectrum for the PLGA scaffold (see Supplementary Materials Figure S4a) is identical to the spectrum in reference [52]. For PLGA scaffolds, 1749 cm^−1^ indicates the stretching of the C=O bond [52,53]. It can be clearly seen that peaks at 1749 cm^−1^ are visible for PLGA and PVA3% samples, but no such peaks are present for PVA6% and PVA9% samples. This may be due to the fact that on PVA6% and PVA9%, a uniform thick PVA layer with thickness from 3 µm was formed (Figure 4b), while such a thick coating was not observed on PVA3% samples (Figure 4b). Peaks at a wavelength of 1081 cm^−1^ indicate the stretching of C–O bonds in PLGA [52]. For all samples dip-coated in a PVA solution, two peaks are present at the wavenumbers 3300 cm^−1^ and 2926 cm^−1^. A broad peak at wavenumber 3300 cm^−1^ indicates the O–H stretching of the hydroxyl group from PVA [54,55], while this peak could also be related to O–H stretching of water [56], which could persist after the dip-coating process (SI Figure S4). Peaks at the wavenumber of 2926 cm^−1^ are related to the stretching of the C–H bonds that are part of the chemical structure of PVA [54,55]. The peak at wavenumber 1420 cm^−1^ indicates the C–H bending vibration of the CH_2_ groups in the PVA polymer backbone [55]. In the case of the PVA3% samples, on which the PVA layer is not clearly visible, the peaks at 1749 cm^−1^ and 1081 cm^−1^ indicate the stretching of the C=O and C–O groups of the PLGA polymer. On samples with thick PVA layers (PVA6% and PVA9%), the wavenumber of 1081 cm^−1^ reflects the C–O stretching vibration of PVA [54,55]. The wavelength at 839 cm^−1^ indicates the C–C vibrational stretching of the PVA polymer backbone [57].

From the thermogravimetric curves, it can be seen that when reaching 100 °C (see Supplementary Materials Figure S5b), approximately 3 to 4% of the mass is lost in all samples, which is attributed to the evaporation of water [58]. When the temperature reaches 800 °C, all samples are completely burnt (Figure S5a,b). The degradation peak of the unmodified PLGA scaffold can be observed at 376.42 °C (see Supplementary Materials Figure S5c). These results are consistent with the literature, as it has been reported that the differential thermogravimetric analysis (DTG) peak of PLGA is present at 380 °C [59]. The degradation peaks for PVA3%, PVA6% and PVA9% are observed at 371.15 °C, 355.43 °C and 330.76 °C, respectively (see Supplementary Materials Figure S5c). A linear decrease in decomposition temperature occurs with increasing mass of PVA in the samples (see Supplementary Materials Figure S6). This may be due to the difference in degradation temperature between PLGA and PVA. The degradation peak for PVA is approximately 319 °C [60], while PLGA with a lactide-to-glycolide ratio of 85/15 has such a peak at about (376–380) °C [59].

From the X-ray diffraction patterns (Figure S4b), it can be concluded that the unmodified PLGA scaffolds are amorphous, which is in agreement with the results reported previously [41]. However, for PVA3% samples, two high-intensity peaks can be observed at 2θ of 14.1° and 16.95°. The positions of the peaks indicate a crystalline PVA film [61] and may indicate the presence of the two polymers PLGA and PVA. It is known from the literature that composite polymer films prepared from silk fibroin and PVA display two crystalline peaks at 2θ of 14.1° and 16.9° [62]. Two peaks at 2θ of 14.1° and 16.95° can also be identified for the PVA6% samples, but their intensity is not as high as for the PVA3% samples. For the PVA9% samples, no peaks were observed that indicate a crystal structure of the PVA coating or the PLGA scaffold. The PVA3% and PVA6% samples have a degree of crystallinity of (77 ± 3)% and (37 ± 1)%, respectively (SI Table S3). The crystallite size for PVA3% and PVA6% samples is (9.5 ± 0.6) and (9.2 ± 0.6) nm, respectively (SI Table S3). It should be noted that the sample with the thinnest PVA coating (PVA3%) has the highest degree of crystallinity at (77 ± 3)%, while the sample with the thickest PVA coating (PVA9%) has an amorphous structure. This might be a consequence of the presence of the two polymers PVA and PLGA, which only interact with each other only at the interface between the PLGA scaffold and the PVA coating. It is therefore possible that the crystalline interface overlaps with the amorphous PVA film as the thickness of the PVA layer increases.

### 3.5. Wettability of the PLGA Scaffold Samples

PLGA scaffolds have hydrophobic properties, and when the contact time of water droplets with the samples increases from 2 s to 60 s, the water contact angle (WCA) does not change and is in the range of ((128.8–129.3) ± 2.2)° (see Supplementary Materials Figure S7). Similar values of the WCA for PLGA scaffolds have already been reported [41]. However, after dip-coating PLGA scaffolds in PVA solutions, the WCA changes significantly and the initially hydrophobic samples become hydrophilic (Figure S7). This is due to the fact that PVA coatings have hydrophilic properties [63]. The WCA after contact of the water droplet with its surface is in the range of ((65.7–67.6) ± 2.6)° for PVA3%, ((62.9–65.0) ± 1.3)° for PVA6% and for ((55.1–56.7) ± 1.6)° for PVA9% (Figure S7). It can be seen that as the contact time of the water droplets with the PVA-coated samples increases from 2 s to 60 s, the WCA weakly decreases in the range of 1° to 2° (see Figure S7a–c). Increasing the viscosity of the PVA solution from 3 wt.% to 9 wt.% reduces the WCA of the dip-coated samples from 9° to 12°. This is related to the fact that the thickness of the hydrophilic PVA coating on the hydrophobic PLGA scaffolds increases with increasing PVA solution viscosity (Figure 4a,b).

### 3.6. Mechanical Properties of the PLGA Scaffolds

The mechanical properties of the unmodified and PVA-coated PLGA scaffolds are presented in see Supplementary Materials Figure S8. Here, the tensile strength of PLGA is (5.33 ± 0.55) MPa, similar values to those already reported in [41]. The tensile strength of PVA3%, PVA6% and PVA9% samples corresponds to (6.25 ± 0.35) MPa, (6.17 ± 0.37) MPa and (7.71 ± 0.82) MPa, respectively (Figure S8). After dip-coating of PLGA scaffolds in PVA solution with 3 wt.% and 6 wt.%, the tensile strength increases by 1.16 to 1.17 times in comparison to the unmodified scaffolds, while the tensile strength increases by 1.45 times after dip-coating in 9 wt.% PVA solution. These results are to be expected, since it is known that the mechanical properties of the scaffolds increase with increasing viscosity of the dip-coating solution [64]. Reference [65] also reports that the mechanical properties of hydroxyapatite scaffolds after dip-coating in PCL solution increase with increasing PCL concentration in trichloromethane.

### 3.7. Cytotoxicity and Anti-Adhesive Properties of the PLGA Scaffold Samples

**Figure 5:**
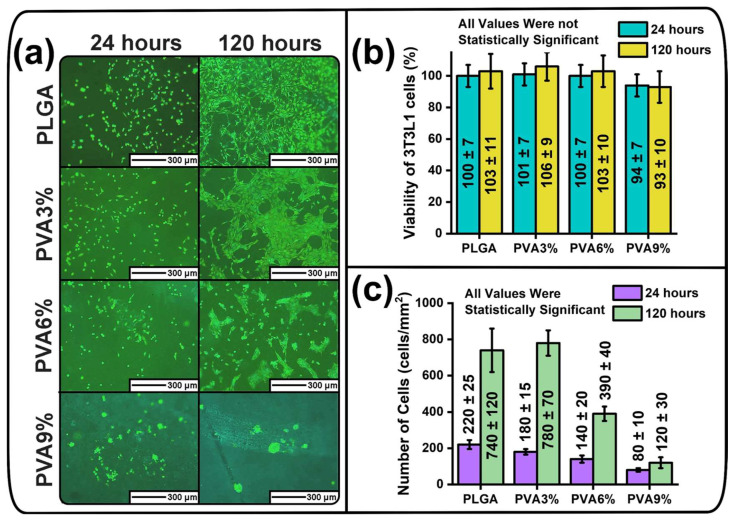
Cell adhesion of mouse fibroblast of the cell line 3T3-L1 on the surface of unmodified (PLGA) and dip-coated (PVA3%, PVA6%, PVA9%) scaffolds and cytotoxicity of sample extracts: (**a**) fluorescent micrographs of mouse fibroblast of the cell line 3T3-L1 visualizing their adhesion to the sample surfaces; (**b**) viability of mouse fibroblast of the cell line 3T3-L1 in the sample extracts indicating the cytotoxic properties of the samples (mean ± standard deviation (M ± SD), n = 12); (**c**) number of cells counted from the fluorescent micrographs (M ± SD, n = 12).

The fluorescent micrographs illustrating the adhesion of mouse fibroblast on the surfaces of the unmodified and dip-coated PLGA scaffold samples, the viability of the fibroblasts in the sample extracts and the number of fibroblasts counted from the fluorescent micrographs are presented in Figure 5.

After 24 h of incubation, it can be seen that the fibroblasts are evenly distributed on the surfaces of the PLGA, PVA3% and PVA6% scaffold samples and do not form clusters (Figure 5a). However, after 24 h of incubation, the PVA9% samples exhibit that the fibroblasts interact with each other and form spherical clusters, as well as being unevenly distributed (Figure 5a). After 120 h of incubation, fluorescence micrographs of the fibroblasts attached to the surface of PLGA and PVA3% scaffold samples indicate that the fibroblasts have lamellipodia and attempt to cover as much surface area as possible, indicating that these samples have no anti-adhesive properties (Figure 5a). In the case of PVA6% samples, after 120 h of incubation, it can be observed that it is difficult for the fibroblasts to spread on the surface of the sample, as evidenced by voids between cell agglomerates and the presence of several islets consisting of 1–5 spherical fibroblasts without lamellipodia (Figure 5a). These results indicate that the PVA6% samples exhibit weak anti-adhesive properties. After 120 h of incubation of the fibroblasts on PVA9% samples, it can be seen that the fibroblasts are unable to anchor to the sample surface. The fibroblasts form spherical three-dimensional agglomerates to minimize the contact area with the surface of the PVA9% samples (Figure 5a). Such agglomerates consist of spherical fibroblasts that do not have lamellipodia. This indicates the high anti-adhesive properties of the PVA9% samples.

The cell viability of mouse fibroblast of the cell line 3T3-L1 is in the range of ((93–106) ± 10)% for all samples (Figure 5b), indicating that the unmodified PLGA scaffolds and the dip-coated scaffolds did not contain cytotoxic compounds. The cytotoxicity data between the unmodified and dip-coated samples, as well as the data at different incubation durations (24 and 120 h), are not statistically significant (Figure 5b). During the four-day incubation (from 24 h to 120 h), the numbers of adhered fibroblasts on the surface of PLGA and PVA3% samples increased from (220 ± 25) to (740 ± 120) cells/mm^2^ and from (180 ± 15) to (780 ± 70) cells/mm^2^, respectively, indicating an increase by approximately 3.4 to 4.3 times (Figure 5c). In the case of the PVA6% sample, the number of adhered fibroblasts increased from (140 ± 20) to (390 ± 40) cells/mm^2^, which indicates an increase by 2.8 times within 4 days, whereas it only increased by 1.5 times (from (80 ± 10) to (120 ± 30) cells/mm^2^) for the PVA9% samples (Figure 5c). The number of adherent cells between all unmodified and dip-coated samples, as well as the number for different incubation times, is statistically significant at *p* < 0.01 (Figure 5c). This statistical difference proves that the minimum number of cells is observed on the PVA9% sample, while the highest number of adhered fibroblasts is present on the PLGA and PVA3% samples.

In accordance with the results of in vitro studies, the PVA9% samples exhibit the most pronounced anti-adhesive properties. The PVA9% samples investigated in this study have prospects for further use in the prevention of adhesion and may be more effective than existing commercial equivalents. For example, the commercial and widely used adhesion prevention membrane Seprafilm^®^ (Deerfield, IL, USA) can be cytotoxic [66], whereas the PVA9% samples are not (Figure 5b). Additionally, the PVA9% samples can reduce the number of adherent NIH3T3 cells by 63.6% and 83.8%, after 24 and 120 h of incubation, respectively (Figure 5c), while the use of commercially available anti-adhesive membrane SurgiWrap^®^ has statistically similar anti-adhesive properties to unmodified PLGA thin films for NIH3T3 cells in vitro, as in [67]. Hydrogels and membranes composed of PVA also show more effective adhesion prevention results in vivo than commercial Seprafilm^®^ (Deerfield, IL, USA) membranes [68].

It can be concluded that the thicker the PVA coatings on PLGA scaffolds, the stronger the anti-adhesive properties. This is confirmed by the fact that PLGA and PVA3% samples, on which PVA coatings were not observed in the bright-field micrographs of the cross-section (Figure 4a), are characterized by a lack of anti-adhesion properties (Figure 5a). At the same time, PVA6% samples with an average PVA coating thickness of (3.32 ± 0.97) µm have weak anti-adhesive properties, and PVA9% samples with the greatest PVA coating thickness of (8.09 ± 1.43) µm (Figure 4b) exhibit the highest anti-adhesive properties (Figure 5a).

These results are in agreement with studies in which smooth PVA and PVA-containing films exhibit low cell adhesion [69,70,71]. The increase of anti-adhesive properties with increasing PVA layer thickness can also be associated with the increase in the degree of hydration caused by the hydroxyl groups of PVA [72]. It should be noted that the anti-adhesive properties increase with increasing degree of hydration [73]. Chen et al. [74] report that antifouling and anti-adhesive properties are mainly caused by surface hydration. The occurrence of spherical three-dimensional fibroblasts agglomerates on the surface of PVA9% samples is in agreement with the literature, in which cell adhesion on PVA composite films has been investigated [72]. These agglomerates are also related to surface hydration [72]. The presence of a hydration layer indicates that individual water molecules cover the surface of the material [75]. Besides PVA, there are a number of polymers with hydrophilic properties whose anti-fouling and anti-adhesive properties are due to the formation of a hydration layer [76,77]. For example, hydrophilic poly(ethylene glycol) and zwitterion polymers possess strong anti-fouling and anti-adhesive properties [78,79], which can be explained by the formation of a hydration layer [79,80]. The formation of a hydration layer on hydrophilic surfaces prevents the uptake of proteins that are responsible for successful cell attachment [28]. Currently, the mechanism which explains how hydration layers prevent surface fouling is still unknown [81]. Nevertheless, a direct correlation between strong hydrogen bonds of water molecules on the surface of polymers and strong anti-fouling properties has been found [82].

It should be noted that in this study, the morphology of the samples has no significant influence on the adhesion of the fibroblasts. This is supported by the optical profilometry results (Figure 2 (morphology) and Figure 3d (roughness)), which show that PVA3% samples have a similar surface morphology and average roughness depth (R_z_) values to the PVA6% and PVA9% samples, but their anti-adhesive properties are substantially different.

It is known that PVA can degrade rapidly in the body, as it is water-soluble. For example, it was reported that micro-needles consisting of PVA were almost undetectable after 6 days following their injection into a mouse [83]. Nevertheless, the presence of an anti-adhesive membrane for 3 to 5 days is sufficient to prevent the formation of adhesions, as the mesothelium regenerates completely during this time [84]. Therefore, rapid degradation of PVA should not be a concern, as soft tissues have a very fast regeneration rate. However, if the rapid degradation of PVA proves to become a disadvantage, its chemical structure can be cross-linked [85]. The purpose of this chemical modification of PVA is to create hydrogen bonds between the hydroxyl groups [86]. A cross-linked medical PVA graft was reported to maintain its tubular shape throughout an experiment in vivo, which was performed within 12 weeks [87]. It should be noted that the chemical cross-linking of PVA preserves its anti-adhesive properties [22,88].

**Figure 6:**
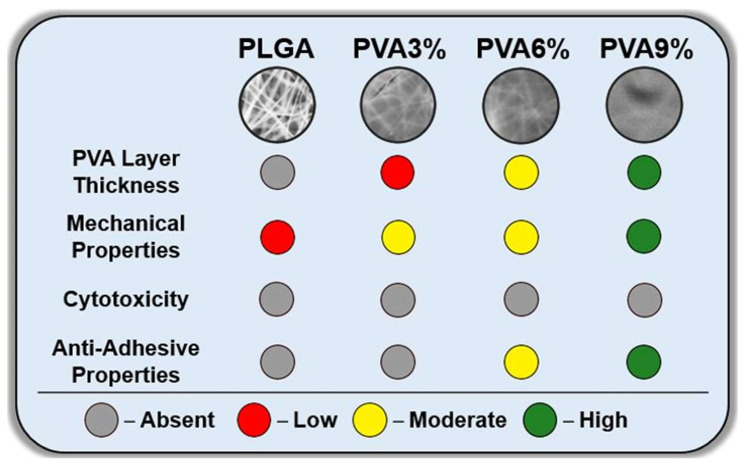
Schematic overview of the investigation results obtained for the unmodified PLGA scaffolds and PVA-coated scaffold samples. The samples are as follows: PLGA—unmodified electrospun PLGA scaffold; PVA3% to PVA9%—PLGA scaffolds dip-coated in aqueous PVA solutions with PVA concentrations of 3 wt.%, 6 wt.% and 9 wt.%.

The general properties of prepared unmodified and dip-coated PLGA scaffolds are shown in Figure 6. After dip-coating, the nonwoven morphology of samples changes to uniform, which indicate the formation of a PVA layer on electrospun scaffolds. With the increase in concentration of PVA in solution, the thickness of the PVA layer increases, as do its mechanical properties (tensile strength, Young’s modulus). All sample extracts are nontoxic towards 3T3-L1 mouse fibroblasts. At the same time, with increasing concentration of polyvinyl alcohol in the solutions, the anti-adhesion properties of the prepared samples towards 3T3-L1 cells increase significantly.

## 4. Conclusions

In this study, polyvinyl alcohol coatings on electrospun poly(lactide-co-glycolide) scaffolds were successfully prepared on the basis of dip-coating in aqueous solutions with different polyvinyl alcohol concentrations of 3 wt.%, 6 wt.% and 9 wt.%. The scanning electron microscope surface micrographs of the samples show that as the polyvinyl alcohol concentration increases, the thickness of the polyvinyl alcohol coatings increases, and the number of cracks on the sample surfaces decreases. These polyvinyl alcohol coatings did not change the mean fiber diameter of the poly(lactide-co-glycolide) scaffolds, indicating that the individual poly(lactide-co-glycolide) fibers did not swell. As the viscosity of the polyvinyl alcohol solution increases, the porosity values of the dip-coated poly(lactide-co-glycolide) scaffolds decrease. The average roughness depth of unmodified poly(lactide-co-glycolide) scaffolds is significantly higher than that of the samples dip-coated in the polyvinyl alcohol solutions. According to the Fourier-transform infrared spectra, the unmodified samples exhibit a characteristic peak indicative of the poly(lactide-co-glycolide) polymer. Scaffolds dip-coated in a solution with a polyvinyl alcohol concentration of 3 wt.% display Fourier-transform infrared spectroscopy peaks corresponding to polyvinyl alcohol and poly(lactide-co-glycolide) polymers. Moreover, in the Fourier-transform infrared spectra of the samples dip-coated in 6 wt.% and 9 wt.% polyvinyl alcohol solutions, only peaks indicative of polyvinyl alcohol are present. As the viscosity of the polyvinyl alcohol solutions increases, a thicker polyvinyl alcohol layer appears on the surface of poly(lactide-co-glycolide) scaffolds. The highest mechanical properties (tensile strength, Young’s modulus) are observed for the sample dip-coated in the most viscous polymer solution with the highest polyvinyl alcohol concentration, while unmodified scaffolds have the lowest mechanical properties. The cytotoxicity assay indicated that all sample extracts possess no cytotoxicity against 3T3-L1 fibroblasts. The adhesion test with 3T3-L1 fibroblasts illustrated that the anti-adhesion properties increased with increasing thickness of polyvinyl alcohol coating on the scaffold surfaces. These findings will be useful for the fabrication of flexible, biocompatible and biodegradable membranes with high anti-adhesive properties for biomedical purposes to prevent adhesions in the intra-abdominal cavity.

## Data Availability

Data underlying the results presented in this paper are not publicly available at this time but may be obtained from the authors upon reasonable request.

## Supplementary Materials

The following supporting information can be downloaded at: https://www.mdpi.com/article/10.3390/jfb16020057/s1. Figure S1. (a) The dynamic viscosity of solutions with 3 wt.%, 6 wt.% and 9 wt.% PVA in distilled water; (b) changes in the weight in milligrams (mg) of PLGA scaffolds after dip-coating in 3 wt.%, 6 wt.% and 9 wt.% PVA solutions; (c) changes in the weight in percentage (%) of the values in (b). Figure S2. (a) Dependence of the PVA concentration in an aqueous solution on its dynamic viscosity. (b) Weight dependence of the dip-coated PLGA scaffolds on the dynamic viscosity of the aqueous PVA solutions. The dynamic viscosity of the aqueous solution without PVA (0 wt.%) is 0.9 mPa∙s. Figure S3. Morphology of the unmodified PLGA scaffolds and the PLGA scaffolds dip-coated in aqueous PVA solution with different concentrations of 3 wt.% (PVA3%), 6 wt.% (PVA6%) and 9 wt.% (PVA9%). BF—bright-field micrographs taken with 10× and 20× objectives; SEM—scanning electron microscope micrographs taken at a magnification of 1000×. Figure S4. FTIR spectra (a) and XRD diffractograms (b) of unmodified PLGA scaffolds (PLGA) and dip-coated PLGA scaffolds in PVA solution with different concentrations of 3 wt.% (PVA3%), 6 wt.% (PVA6%) and 9 wt.% (PVA9%). Figure S5. Thermal analysis results of unmodified PLGA scaffolds (PLGA) and dip-coated PLGA scaffolds in solution with different PVA concentrations of 3 wt.% (PVA3%), 6 wt.% (PVA6%) and 9 wt.% (PVA9%): (a) thermogravimetric analysis (TGA) curves representing the mass loss in mg with increasing temperature; (b) thermogravimetric analysis (TGA) curves representing the mass loss in % with increasing temperature; (c) differential thermogravimetric analysis (DTG) curves representing the rate of mass loss in mg/min with increasing temperature; (d) differential thermogravimetric analysis (DTG) curves representing the rate of mass loss in %/min with increasing temperature. Figure S6. Correlation between the increase in sample mass (Figure S1c) and the degradation peaks of the samples determined in DTG curves (Figure S5c). Figure S7. Water contact angle (WCA) values at different contact times of water droplets with the surface of the unmodified (PLGA) and dip-coated (PVA3%, PVA6% and PVA9%) sample scaffolds: (a) WCA after 2 s of contact; (b) WCA after 30 s of contact; (c) WCA after 60 s of contact. Figure S8. Mechanical properties of unmodified PLGA scaffolds (PLGA) and dip-coated scaffold samples in aqueous PVA solutions at different wt.% concentrations (PVA3%, PVA6% and PVA9%): (a) tensile strength; (b) maximum elongation at break; (c) Young’s modulus. Table S1. Initial mass of the scaffold samples. Table S2. Elemental composition and elemental ratio of unmodified PLGA scaffolds (PLGA) and dip-coated scaffold samples in aqueous PVA solutions at different wt.% concentrations (PVA3%, PVA6% and PVA9%). The elemental composition (determined by energy-dispersive X-ray spectroscopy (EDX)) is indicated in weight (wt.%) and atomic (at.%) percent. Table S3. Crystallinity degree and mean size of the crystallites of the samples under investigation according to the results of the XRD measurements (Figure S4b).
