# Supplementary material for: Fabrication of PVA Coatings Applied to Electrospun PLGA Scaffolds to Prevent Postoperative Adhesions
Source: J Funct Biomater. 2025 Feb 10;16(2):57. doi: 10.3390/jfb16020057 (PMC11856736; doi:10.3390/jfb16020057)
Supplement: Supplementary file 1 [file jfb-16-00057-s001.zip › jfb-3423471-supplementary.pdf]

Journal of
Functional Biomaterials

Article Support Information

Support Information: Fabrication of PVA coatings applied to electrospun PLGA scaffolds to prevent postoperative peritoneal adhesions

Arsalan D. Badaraev [1], Evgenii V. Plotnikov [2,3], Vladislav R. Bukal [1], Gleb E. Dubinenko [1], Johannes Frueh [1], Sven Rutkowski [1,*] and Sergei I. Tverdokhlebov [1,*]

[1] Weinberg Research Center, School of Nuclear Science & Engineering, National Research Tomsk Polytechnic University, 30, Lenin Avenue, 634050, Tomsk, Russia; adb6@tpu.ru (A.D.B.); vrb2@tpu.ru (V.R.B.); dubinenko@tpu.ru (G.E.D.); johannes.frueh@alumni.ethz.ch (J.F.)
[2] Research School of Chemistry & Applied Biomedical Sciences, Tomsk Polytechnic University, 634000 Tomsk, Russia; plotnikovev@tpu.ru (E.V.P.)
[3] Mental Health Research Institute, Tomsk National Research Medical Center of the Russian Academy of Sciences, Aleutskaya Street, 634014 Tomsk, Russia; plotnikovev@tpu.ru (E.V.P.)
* Correspondence: rutkowski_s@tpu.ru (S.R.); tverd@tpu.ru (S.I.T.)

1. Supplementary Figures

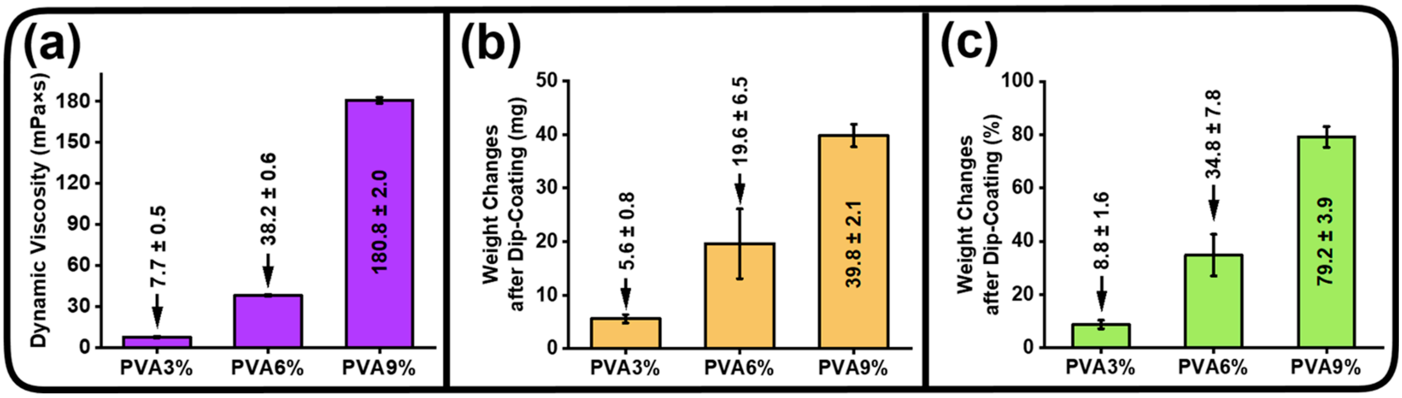

Figure S1. (a) The dynamic viscosity of solutions with 3 wt.%, 6 wt.% and 9 wt.% PVA in distilled water; (b) changes in the weight in milligrams (mg) of PLGA scaffolds after dip-coating in 3 wt.%, 6 wt.% and 9 wt.% PVA solutions; (c) changes in the weight in percentage (%) of the values in (b).

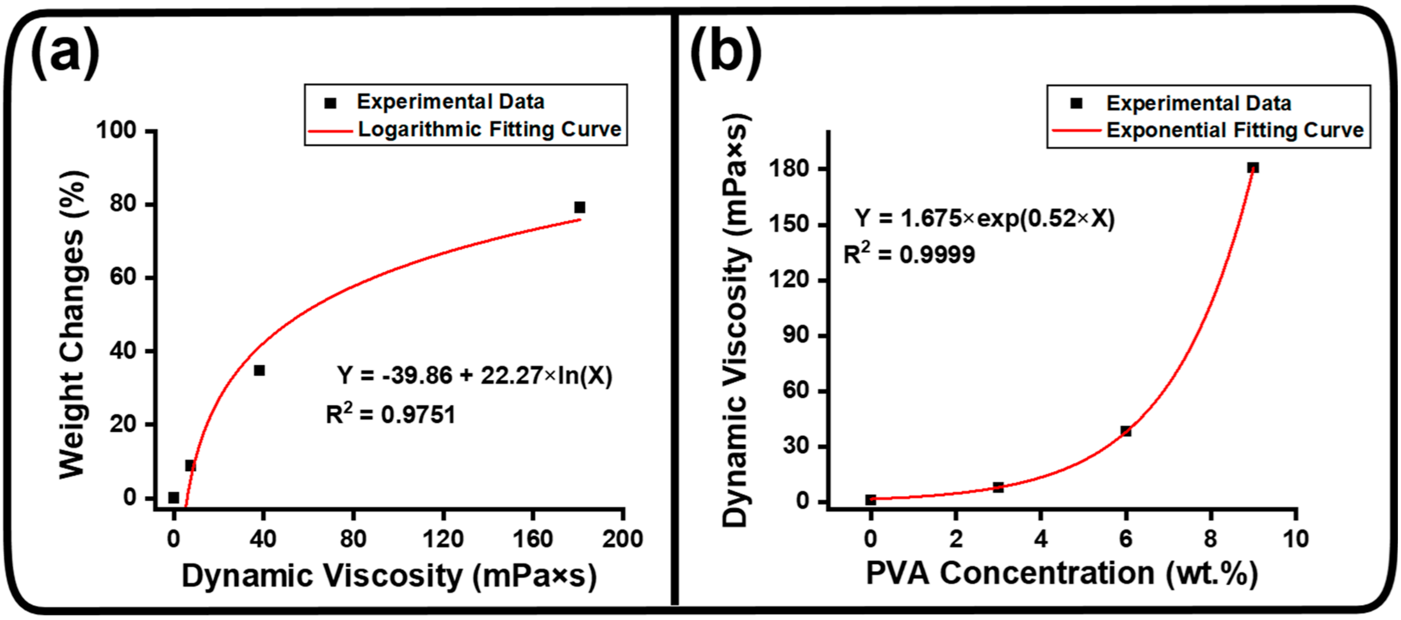

Figure S2. (a) Dependence of the PVA concentration in an aqueous solution on its dynamic viscosity. (b) Weight dependence of the dip-coated PLGA scaffolds on the dynamic viscosity of the aqueous PVA solutions. The dynamic viscosity of the aqueous solution without PVA (0 wt.%) is 0.9 mPa×s.

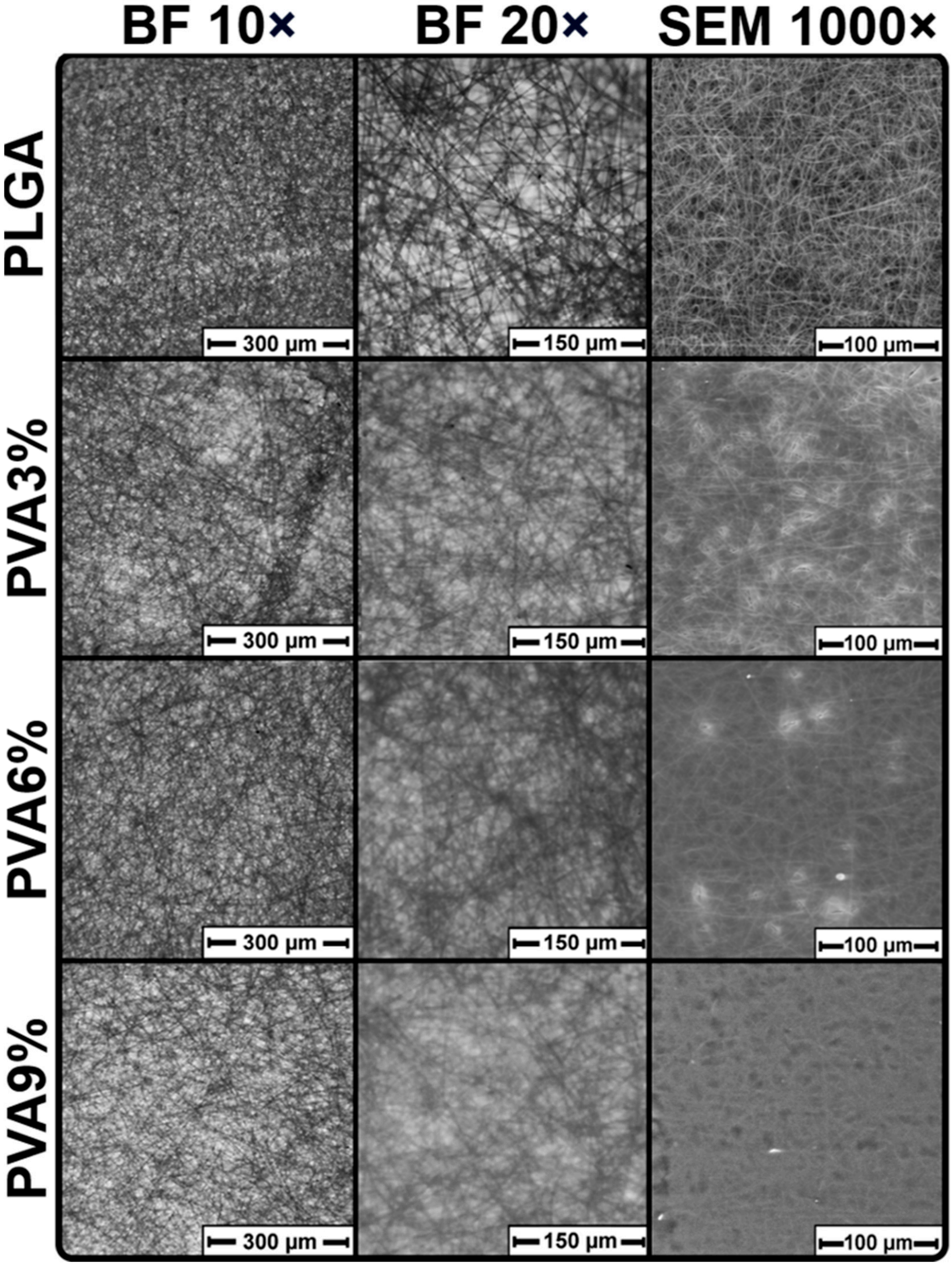

Figure S3. Morphology of the unmodified PLGA scaffolds and the PLGA scaffolds dip-coated in aqueous PVA solution with different concentrations of 3 wt.% (PVA3%), 6 wt.% (PVA6%) and 9 wt.% (PVA9%). BF—bright-field micrographs taken with 10× and 20× objectives; SEM—scanning electron microscope micrographs taken at a magnification of 1000×.

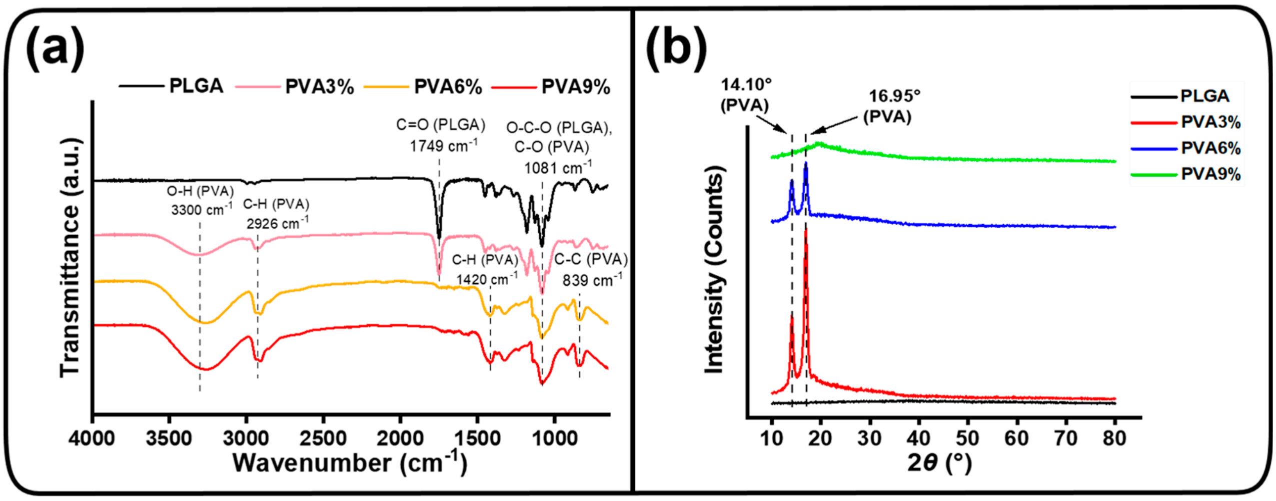

Figure S4. (a) FTIR spectra and (b) XRD diffractograms of unmodified PLGA scaffolds (PLGA) and dip-coated PLGA scaffolds in PVA solution with different concentration of 3 wt.% (PVA3%), 6 wt.% (PVA6%) and 9 wt.% (PVA9%).

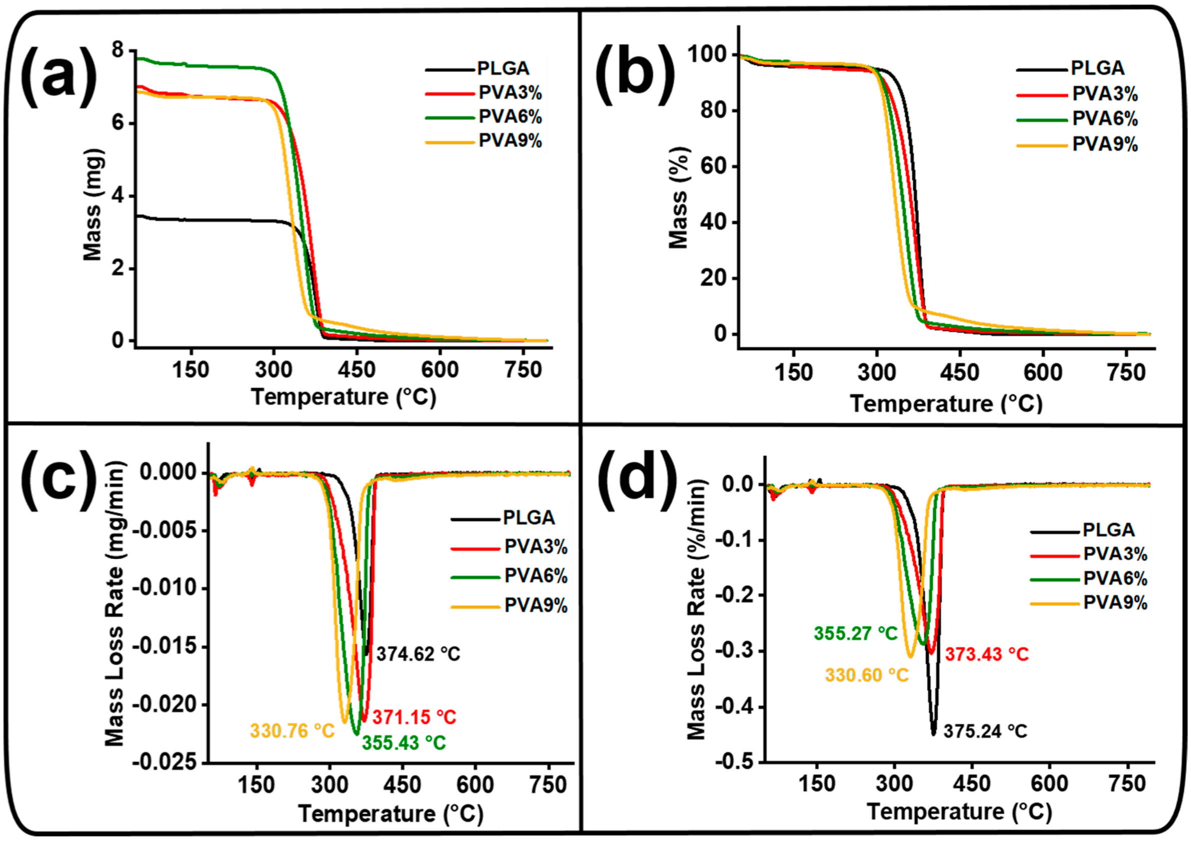

Figure S5. Thermal analysis results of unmodified PLGA scaffolds (PLGA) and dip-coated PLGA scaffolds in solution with different PVA concentrations of 3 wt.% (PVA3%), 6 wt.% (PVA6%) and 9 wt.% (PVA9%): (a) thermogravimetric analysis (TGA) curves representing the mass loss in mg with increasing temperature; (b) thermogravimetric analysis (TGA) curves representing the mass loss in % with increasing temperature; (c) differential thermogravimetric analysis (DTG) curves representing the rate of mass loss in mg/min with increasing temperature; (d) differential thermogravimetric analysis (DTG) curves representing the rate of mass loss in %/min with increasing temperature.

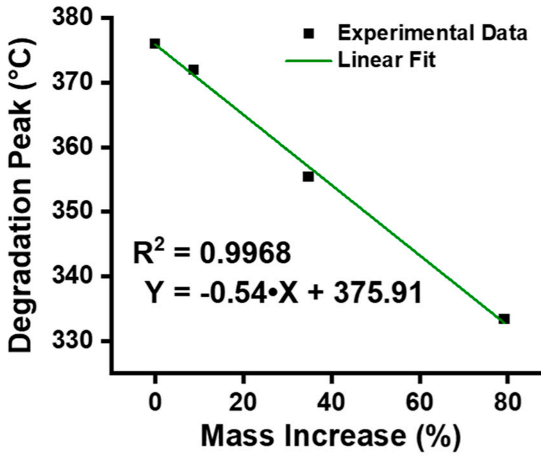

Figure S6. Correlation between the increase in sample mass (Figure S1c) and the degradation peaks of the samples determined in DTG curves (Figure S5c).

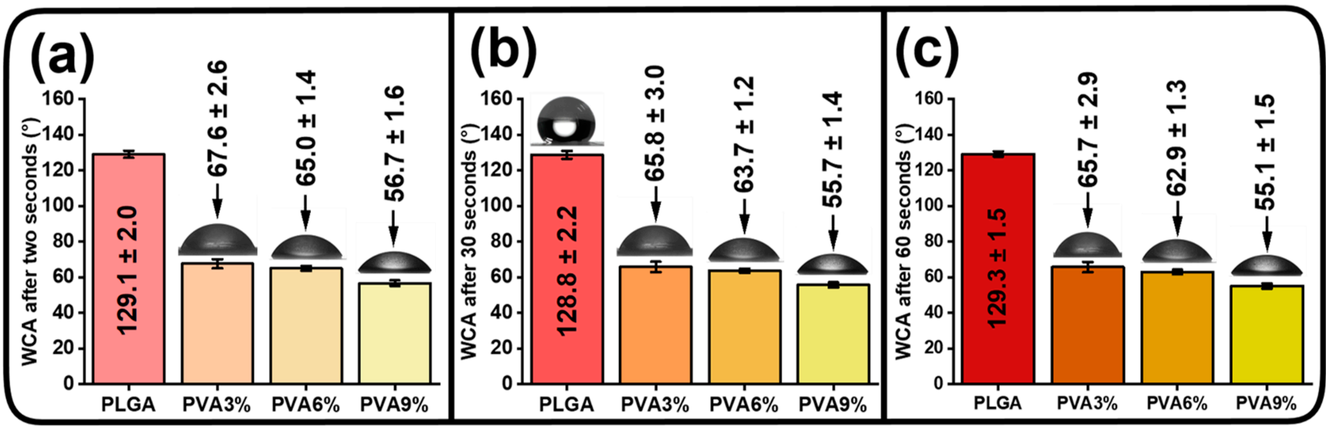

Figure S7. Water contact angle (WCA) values at different contact times of water droplets with the surface of the unmodified (PLGA) and dip-coated (PVA3%, PVA6% and PVA9%) sample scaffolds: (a) WCA after 2 seconds of contact; (b) WCA after 30 seconds of contact; (c) WCA after 60 seconds of contact.

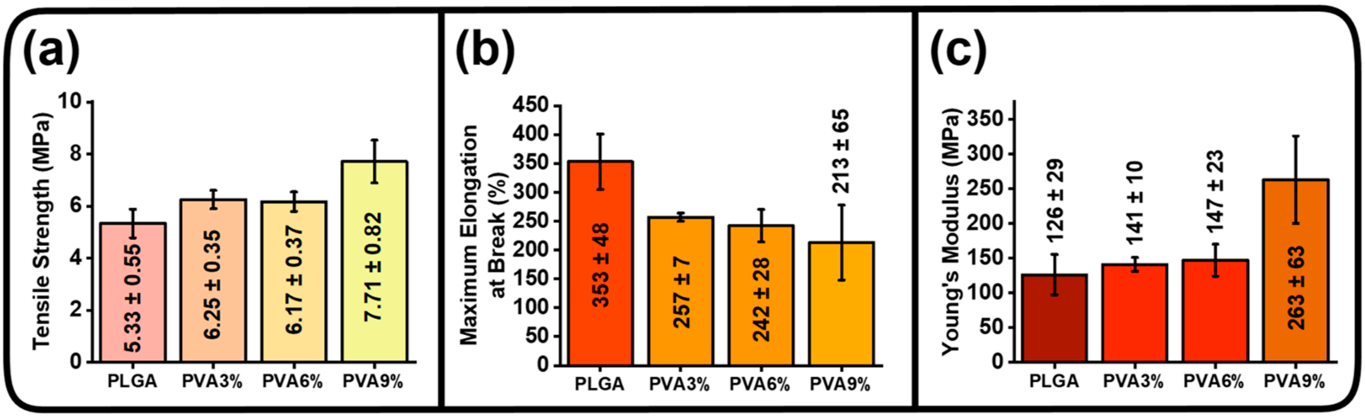

Figure S8. Mechanical properties of unmodified PLGA scaffolds (PLGA) and dip-coated scaffold samples in aqueous PVA solutions at different wt.% concentrations (PVA3%, PVA6% and PVA9%): (a) tensile strength; (b) maximum elongation at break; (c) Young's modulus.

2. Supplementary Tables

Table S1. Initial mass of the scaffolds samples.

Sample	Initial Mass (mg)
PVA3%	64.0±3.4
PVA6%	57.0±9.2
PVA9%	50.0±2.1

Table S2. Elemental composition and elemental ratio of unmodified PLGA scaffolds (PLGA) and dip-coated scaffold samples in aqueous PVA solutions at different wt.% concentrations (PVA3%, PVA6% and PVA9%). The elemental composition (determined by energy-dispersive X-ray spectroscopy (EDX)) is indicated in weight (wt.%) and atomic (at.%) percent.

Sample	Elemental Composition				Elemental Ratio	
	wt.%		at.%		wt.%	at.%
at	Carbon (C)	Oxygen (O)	Carbon (C)	Oxygen (O)	C/O	C/O
PLGA	56.8±0.1	43.2±0.1	63.6±0.1	36.4±0.1	1.31	1.74
PVA3%	59.4±0.1	40.6±0.1	66.1±0.1	33.9±0.1	1.46	1.95
PVA6%	60.5±0.1	39.5±0.1	67.0±0.1	33.0±0.1	1.53	2.03
PVA9%	62.9±0.1	37.1±0.1	69.3±0.1	30.7±0.1	1.70	2.26

Table S3. Crystallinity degree and mean size of the crystallites of the samples under investigation according to the results of the XRD measurements (Figure S4b).

Sample	Crystallinity Degree (°)	Mean Size of Crystallites (nm)
PLGA	-	-
PVA3%	77±3	9.5±0.6
PVA6%	37±1	9.2±0.6
PVA9%	-	-
